# Pharmacists are initiators in palliative care for patients with rare diseases

**DOI:** 10.1186/s13023-023-02765-8

**Published:** 2023-06-08

**Authors:** M. Dooms

**Affiliations:** University Hospitals Leuven, Leuven, Belgium

**Keywords:** Rare diseases, Orphan drugs, Palliative care, Pharmaceutical care, Medication review, Deprescribing

## Abstract

The World Health Organization supports early delivery of palliative care as it reduces unnecessary hospital admissions and the inappropriate use of health care services. A community pharmacist can play a key role in advocating timely access to palliative care. Medication reconciliation must alert them to start communicating with the patient and/or his relatives about refocusing treatment and care as part of palliative and terminal care. Pharmaceutical activities for these patients include dispensing of devices and medicinal products, compounding personalized medication and participating as a member of the Palliative Support Team. Most of the several thousands of rare diseases are caused by genetic defects and up to now have no cure and a late diagnosis.

## Methodology

A “rare disease” in this context is a life-threatening or chronically debilitating condition with a low prevalence. The majority of these disorders have a genetic origin and up to now no chance for cure. Symptom treatment and (para)medical care can improve the quality of life and extend life expectancy. Examples of rare diseases in children that would benefit from palliative care [1–7] are Batten disease, Duchenne Muscular Dystrophy [8], Ehlers-Danlos syndrome, Gaucher’s disease, Krabbe and Pompe disease. For rare lung diseases such as idiopathic pulmonary fibrosis [9] and pulmonary arterial hypertension [10, 11] guidelines on palliative care have been published but the degree of availability of end-of-life care in different countries is often highly variable [12]. Also palliative care plans for patients with Amyotrophic Lateral Sclerosis, also called “motor neuron disease” [13] and other rare long-term degenerative nerve diseases [14] have been proposed. An online Palliative Care Formulary is available [15] and a textbook for use in pediatrics [16] as well as an online instruction course [17] with a Syringe Driver Database. A mobile application is developed for cancer pain management and opioid conversion in the palliative setting [18]. Palliative care procedures for pharmacists need to adhere to these well established guidelines.

Also for rare treatable hematologic malignancies [19] such as Acute Myeloid Leukemia [20] and Chronic Myeloid Leukemia [21] palliative care plans have been published. More recent publications appeared in the literature about palliative care for rare cancers with an orphan drug authorization such as multiple myeloma [22, 23], rare lymphomas [24], myelodysplastic syndromes [25] and soft tissue sarcomas [26]. Most of the rare cancers have no authorized treatment and need palliative and end-of-life care.

**Fig. 1:**
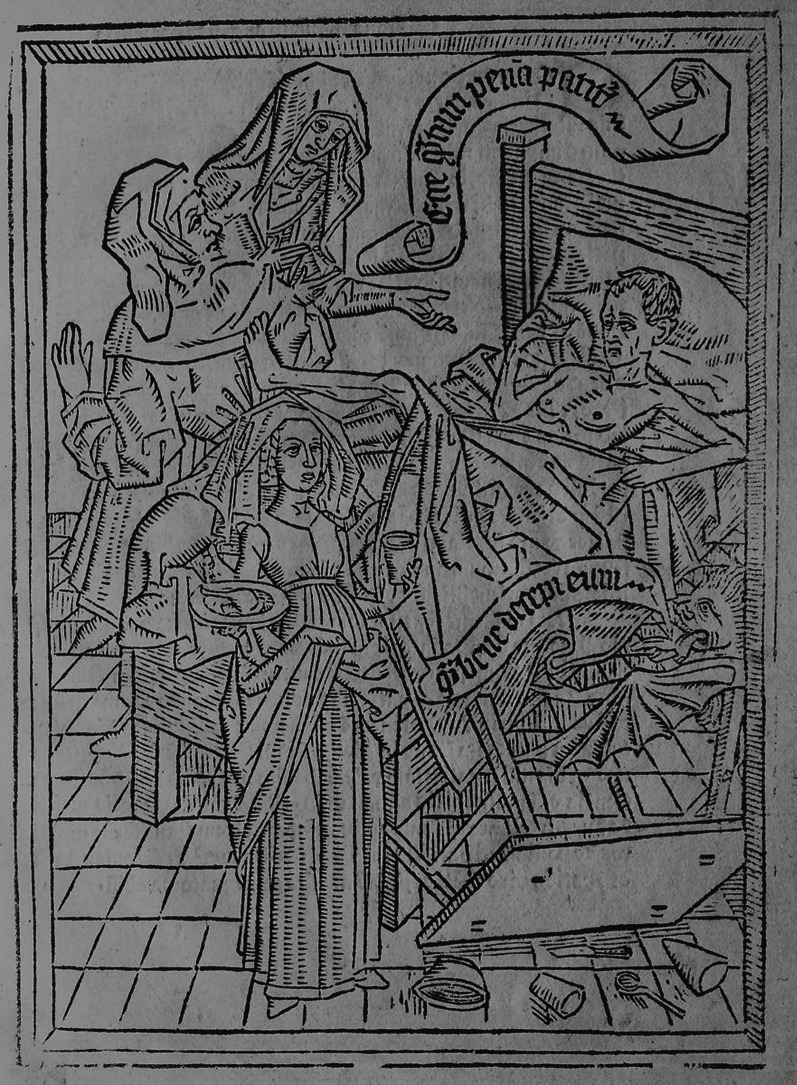
Ars Moriendi (1475) Published by Nicolaus Gotz. Woodcut

Today community pharmacists [27–30], capable and trained to treat patients at a personal level, as well as hospital [31–34] pharmacists are quite late involved in palliative care management practiced for many centuries (Fig. 1). First a structured medication review has to be performed leading to deprescribing all not actually necessary drugs for disease and symptom control (all preventive medication such as statins, bisphosphonates, …) and especially orphan drugs as they have a major budget impact [35]. All community pharmacists in Belgium work with a nationwide shared pharmaceutical file (“Gedeeld Farmaceutisch Dossier”/”Dossier Pharmaceutique Partagé”) and are fully informed about the usage of all the medicines and para-pharmaceutical products for their patients. Dispensing devices (probes, syringe driver, oxygen cannulas) and pain medication, compounding oral liquids and cassettes with morphine hydro chloride are standard pharmaceutical procedures in palliative home care. Pharmacists in nursing homes and hospitals dispense all necessary medication (including “just in case” and anticipatory medication), for subcutaneous intermittent or intravenous continuous (syringe driver) palliative sedation with midazolam and clotiapine [36]. As constipation is a common side-effect of opioids, laxatives are always administered, prescription free [37]. Opioid, antipsychotic and hypnotic drug use in palliative care has been studied in several European countries [38, 39]. Oral care needs always full attention, especially with oxygen therapy. Pharmaceutical education has come a long way to adapt to the needs of the dying patient (Table 1). Many schools of pharmacy have included palliative care in their curriculum what was not the case several years ago.

**Table 1:** End-of-Life and Palliative Care Topics Covered in the Curriculum of US Pharmacy Schools in 2012

Quality of life 94%
Communication with individuals with terminal illnesses 83%
Topic Percentage Covering Attitudes toward death and dying 77%
Communication with family members of patients with terminal illnesses 71%
Psychological aspects of dying (eg, anxiety, depression) 71%
The range of settings including home care, nursing homes, and hospice 69%
The impact of ethnic, religious, and cultural differences 67%
Psycho social interventions to alleviate pain across the life cycle 65%
Grief and bereavement 62%
Advance directives (living will, power of attorney for health care) 60%
Illness-related issues such as decision making in dying and death 60%
Social contexts of dying (eg, family care) 58%
Euthanasia 54%
The physical and multidimensional stages of the dying process 52%
The needs of special populations (eg, children and those with disabilities) 50%
Suicide 44%
Socioeconomic dimensions of patients with terminal illnesses and their families 42%

(Dickinson GE. End-of-Life and Palliative Care Education in US Pharmacy Schools. [cited 2018

Nov 18]; Available from: https://journals-sagepub.com.kuleuven.ezproxy.kuleuven.be/doi/pdf/10.1177/1049909112457011)

Being a member of the Palliative Support Team means proposing alternatives by drug shortages (midazolam) and giving advice by crushing tablets/cutting patches (buprenorphine, fentanyl). Databases around these issues are freely accessible for drug shortages [40] and cutting and crushing issues [41]. Further pharmaceutical care includes looking for alternative ways of administering medicinal products (haloperidol, scopolamine, alizapride) when oral intake becomes difficult and avoiding drug interactions in infusion pump solutions. As more patients prefer to end their life at home, a timely contact with the local community pharmacist is important mainly to avoid misunderstandings on high doses of the pain medication and timely access to medication and devices. As patients become increasingly unwell, they may require frequent and irregular changes to medication regimes in the home care and need considerable support, often from family members, in order to cope with the management of their medications. In Belgium a “palliative lump-sum” is available in the last 2 months of life to decrease the overall personal medical and care costs in the home care [42]. Palliative care networks are installed throughout the country to support palliative home care, also for children. You cannot always cure but you can always care.

In 2021, this is 19 years after the publication of the Belgian euthanasia law (May 2002), 2 699 patients were registered in an euthanasia procedure as foreseen in this law: 54,3% of the procedures were executed at home; 67,8% of the patients was older than 70 y.; 40,2% older than 80 y.; only 1,4% younger than 40 y. Standard operating procedures for the compounding of the necessary infusions were validated and followed for all these patients. Oral administration is avoided: the old “Brompton cocktail” (morphine or diacetylmorphine, cocaine, chlorpromazine, ethyl alcohol) is never used anymore.

As medicinal products for patients with rare diseases are usually expensive, some countries consider the re-dispensing of unused medication returned by one patient for use by another [43, 44]. This can help by reducing the environmental burden and save money in the health care system but most pharmacists are not in favor of such a procedure as they cannot take responsibility for products kept outside the pharmacy for some time (cold chain).

Some of these medications are cytotoxic or narcotic and need correct handling [45, 46]. Family members may bring the medication back to the pharmacy for correct disposal. The collected cytotoxic risk waste is incinerated in household waste incinerators at cost of the pharmaceutical industry.

Last but not least, community and hospital pharmacists know that we live in a multicultural world and some pharmaceutical interventions to be taken are negotiable (time of administration of medicinal products during religious periods) but others are not (dose of a medicine to be administrated). Checklists are available to make legal and financial plans for palliative and end-of-life healthcare in the future [47]. Whatever the preferences are of the families of the palliative patient, correct palliative and terminal care needs to be delivered by the medical as well as the pharmaceutical staff.

## Conclusions

As a first-line health care professional who is easily reachable and present in the long disease trajectories of many patients, the community pharmacist [48] is an ideal caregiver to advocate and support timely in the disease trajectory palliative and terminal care. It reduces unnecessary hospital admissions and the inappropriate use of health care services [49]. European [50] as well as International Associations [51–53] offer all the necessary information on the internet for correct pharmaceutical care in a palliative setting.

## Data Availability

Not applicable.
